# Supplementary material for: A new approach to measuring absolute pitch on a psychometric theory of isolated pitch perception: Is it disentangling specific groups or capturing a continuous ability?
Source: PLoS One. 2021 Feb 22;16(2):e0247473. doi: 10.1371/journal.pone.0247473 (PMC7899369; doi:10.1371/journal.pone.0247473)
Supplement: S1 File — (DOCX) [file pone.0247473.s002.docx]

**A new approach to measuring absolute pitch on a psychometric theory of Isolated Pitch Perception: Is it disentangling specific groups or capturing a continuous ability?**

Nayana Di Giuseppe Germano, Hugo Cogo-Moreira, Fausto Coutinho-Lourenço, Graziela Bortz

List of abbreviations

AIC - Akaike Information Criteria

AIPWR - Ability to identify Isolated Pitch Without Reference

AP – Absolute Pitch

BIC - Bayesian Information Criteria

df S-X² - Degrees of freedom S-chi-square

IRT – Item Response Theory

LCA - Latent Class Analysis

LMR - Likelihood Mendell Rubin

LVM - Latent Variable Model

MLR - Maximum Likelihood Robust

RMSEA - Root Mean Square Error of Approximation

RP – Relative Pitch

SE - Standard Error

SSABIC - Simple Size Adjusted Bayesian Information Criterion

S-X² - S-chi-square

VLMR LRT – Vuong-LO-Mendell-Rubin Likelihood Ratio Test
