# Supplementary material for: A new approach to measuring absolute pitch on a psychometric theory of isolated pitch perception: Is it disentangling specific groups or capturing a continuous ability?
Source: PLoS One. 2021 Feb 22;16(2):e0247473. doi: 10.1371/journal.pone.0247473 (PMC7899369; doi:10.1371/journal.pone.0247473)
Supplement: Attachment — Submitted filename: response_to_reviwers.docx [file pone.0247473.s003.docx]

**PONE-D-20-04410
Absolute Pitch as a Latent Trait
PLOS ONE

Dear Dra. Germano,

Thank you for submitting your manuscript to PLOS ONE. After careful consideration, we feel that it has merit but does not fully meet PLOS ONE’s publication criteria as it currently stands. Therefore, we invite you to submit a revised version of the manuscript that addresses the points raised during the review process.**

**I found the manuscript interesting in that it tries to evaluate if absolute pitch is a trait or a categorical variable. The choice of methodology is appropriate, but the reporting is lacking in quality. The comments from the two reviewers are very constructive and should enable you to improve the manuscript.**

**Answer:** Dear Editor, we are thankful for the opportunity to answer the insightful comments we received. We also would like to state that for solving one of the issues, we needed extra help, which was provided by a newly added co-author, Fausto Lourenco Coutinho. He was fundamental to answer and deal with some issues involving R.

**We would appreciate receiving your revised manuscript by Jun 15 2020 11:59PM. When you are ready to submit your revision, log on to https://www.editorialmanager.com/pone/ and select the 'Submissions Needing Revision' folder to locate your manuscript file.

If you would like to make changes to your financial disclosure, please include your updated statement in your cover letter.

To enhance the reproducibility of your results, we recommend that if applicable you deposit your laboratory protocols in protocols.io, where a protocol can be assigned its own identifier (DOI) such that it can be cited independently in the future. For instructions see:** [**http://journals.plos.org/plosone/s/submission-guidelines#loc-laboratory-protocols**](http://journals.plos.org/plosone/s/submission-guidelines#loc-laboratory-protocols) **Please include the following items when submitting your revised manuscript:**

- **A rebuttal letter that responds to each point raised by the academic editor and reviewer(s). This letter should be uploaded as separate file and labeled 'Response to Reviewers'.**
- **A marked-up copy of your manuscript that highlights changes made to the original version. This file should be uploaded as separate file and labeled 'Revised Manuscript with Track Changes'.**
- **An unmarked version of your revised paper without tracked changes. This file should be uploaded as separate file and labeled 'Manuscript'.**

**Please note while forming your response, if your article is accepted, you may have the opportunity to make the peer review history publicly available. The record will include editor decision letters (with reviews) and your responses to reviewer comments. If eligible, we will contact you to opt in or out.**

**We look forward to receiving your revised manuscript.

Kind regards,

Karl Bang Christensen, Ph.D.
Academic Editor
PLOS ONE**

**Additional Editor Comments:**

**Please provide a revised version addressing the comments from the two reviewers, who are very constructive and have provided comments that should enable you to improve the manuscript. The choice of methodology is appropriate, but the reporting is lacking in quality. You must address test of validity much more rigorously in a revised version. Evaluate fit of a one-dimensional CFA model (reporting chi-square, df and P-value). If you also waht to report the RMSEA with corresponding confidence interval or other indeces of close fit that is OK.**

**Answer:** We thank you for the opportunity to answer all the issues raised by the referees. An important detail regarding our IRT models: differently of Mplus default estimator operating under WLSMV (probit link function), which generates CFI, TLI, RMSEA, we are using a different estimator called robust Maximum-likelihood estimator, which does not generate, under dichotomous items, those above cited global model fit indices. To improve the quality of our report, we did extra data analysis (as suggested by the Reviewer#1) and the results are detailed in the following answers. In order to conduct the extra analysis and interpretation, because they involved R, we invited Fausto Lourenco Coutinho, who is expert in R and a PHD student supervised by Hugo Cogo-Moreira, to join us in this manuscript.

**The figures are attached on their own with no legends or titles. Some of them are not even mentioned in the main text of the paper. One example line 145 states 'fig. 1', but the next line appears to discuss fig. 2?**

**Answer:** Legends and better descriptions regarding the tables were provided.

**Journal Requirements: When submitting your revision, we need you to address these additional requirements.**

**1. Please ensure that your manuscript meets PLOS ONE's style requirements, including those for file naming. The PLOS ONE style templates can be found at:**

**https://journals.plos.org/plosone/s/file?id=wjVg/PLOSOne_formatting_sample_main_body.pdf and**

**https://journals.plos.org/plosone/s/file?id=ba62/PLOSOne_formatting_sample_title_authors_affiliations.pdf**

**Answer:** The manuscript was adjusted in order to meet PLOS ONE’s style requirements. We appreciated the links provided.

**2. Please modify the title to ensure that it is meeting PLOS’ guidelines (https://journals.plos.org/plosone/s/submission-guidelines#loc-title).**

**In particular, the title should be "specific, descriptive, concise, and comprehensible to readers outside the field" and in this case it is not informative and specific about your study's scope and methodology.  When modifying the title please be sure to amend both the title on the online submission form (via Edit Submission) and the title in the manuscript so that they are identical.**

**Answer:** We changed the title to a more specific and descriptive version, which should be more comprehensible to readers outside the field.

**3. Your ethics statement must appear in the Methods section of your manuscript. If your ethics statement is written in any section besides the Methods, please move it to the Methods section and delete it from any other section. Please also ensure that your ethics statement is included in your manuscript, as the ethics section of your online submission will not be published alongside your manuscript.**

**Answer:** The ethics statement is now located at “Participants and study design” subsection (line 113).

[Note: HTML markup is below. Please do not edit.]

Reviewers' comments:

Reviewer's Responses to Questions

**Comments to the Author**

**1. Is the manuscript technically sound, and do the data support the conclusions?

The manuscript must describe a technically sound piece of scientific research with data that supports the conclusions. Experiments must have been conducted rigorously, with appropriate controls, replication, and sample sizes. The conclusions must be drawn appropriately based on the data presented.

Reviewer #1: Partly

Reviewer #2: No**

**2. Has the statistical analysis been performed appropriately and rigorously?

Reviewer #1: No

Reviewer #2: No**

**3. Have the authors made all data underlying the findings in their manuscript fully available?

The** [**PLOS Data policy**](http://www.plosone.org/static/policies.action#sharing) **requires authors to make all data underlying the findings described in their manuscript fully available without restriction, with rare exception (please refer to the Data Availability Statement in the manuscript PDF file). The data should be provided as part of the manuscript or its supporting information, or deposited to a public repository. For example, in addition to summary statistics, the data points behind means, medians and variance measures should be available. If there are restrictions on publicly sharing data—e.g. participant privacy or use of data from a third party—those must be specified.

Reviewer #1: No

Reviewer #2: No**

**Answer:** The data are now provided as supporting information.

**4. Is the manuscript presented in an intelligible fashion and written in standard English?

PLOS ONE does not copyedit accepted manuscripts, so the language in submitted articles must be clear, correct, and unambiguous. Any typographical or grammatical errors should be corrected at revision, so please note any specific errors here.

Reviewer #1: Yes

Reviewer #2: No**

**Answer:** The first manuscript was revised by a professional English-language editing service. The new version of the manuscript was also fully revised by another professional English-language editing service.

**5. Review Comments to the Author

Please use the space provided to explain your answers to the questions above. You may also include additional comments for the author, including concerns about dual publication, research ethics, or publication ethics. (Please upload your review as an attachment if it exceeds 20,000 characters)

Reviewer #1: PONE-D-20-04410

This is an interesting application of modern psychometric methods. The comparison of IRT models and latent class models appear well suited for the theoretical problem posed. I have some suggestions for the data analysis and presentation of results.**

**Answer:** we thank you for all the provided suggestions. They were insightful and contributed for the work to improve significantly.

**1. You should present some basic descriptive information, e.g. the** **frequency distribution for each item (e.g. Perfect, Imperfect, Wrong), and the** **frequency distribution of a simple sum of the items (or two sums according to your two approaches). This allows the reader to get some sense of your data.**

**Answer:** we agree with the reviewer and we are thankful for such the suggestion. More information can be found at page 11 Table 1, which provides the percentage of correct answers for Perfect and Imperfect approach for each item, and at page 12 Table 2, which provides the simple correct answers sum for each item for Perfect and Imperfect approach.

**2. You estimate a 2-parameter and a 3-parameter IRT model. However, it is unclear which of these models you deem is having the best fit. Also, it was unclear to me, which of these models you compare with the latent class model and for which IRT model you present the score distribution in figure 5. Please clarify.**

**Answer:** We followed your recommendation and we are now using AIC/BIC + likelihood test for difference. Regarding the second raised issue, because there are two criteria for rating, the model comparisons between IRT and latent class analysis are always conducted within the same rate criterion. In the revised manuscript, at page 17, it might be read as follows:

“In terms of a model fit, Table 5 depicts the model fit for IRT models (two and three items parameters) for perfect and imperfect rating. Considering the perfect approach, the lowest BIC was in favor of an IRT with two parameters, whereas for the imperfect approach the lowest BIC was in favor of an IRT with three parameters”

**3. Fit is evaluated through a global chi-square test. However, chi-square tests with app. 1000 DF are not optimal. You report χ2(991) = 6635.882, p-value =0.999 (line 239). This must be a typo, if the chisq value is correct, p=0. I suggest you do two things.

3a. For global tests of fit and comparisons of the 2-P and 3-P models, use AIC and BIC as in your comparison with latent class models.**

**Answer:** We agree and reinforce that the comparison was conducted based on AIC and BIC, although the other fit is still reported.

**3b. Evaluate item level fit, e.g. by the item fit tests suggested by Orlando and Thissen (Applied Psychological Measurement 2000). Such tests are available in the IRTPRO software and the free R package mirt (https://cran.r-project.org/web/packages/mirt/mirt.pdf). Such item based fit test may identify some stimuli that are not well modeled by IRT.**

**Answer:** We deeply appreciated the idea about showing the item level fit. We calculated them as described by Orlando and Thissen (2000) using R MIRT package. We added table 4 and a set of figures containing empirical plots for all the items under perfect pitch approach working as illustration. Given these new add-ons, the following information were inserted subheading for dimensional solution (pages 15-16):

“Under IRT with two parameters, imperfect rating indicated that all the items had a good fit (all the S-X² p-values >0.05), whereas, under the perfect approach, two out of the ten items were statistically significant (i.e., items c and d). Under IRT with three parameters, it can be observed that the majority of the items displayed a reduction in their p-values when compared to two parameters, for both approaches.

One reason why the imperfect approach under two parameters, in terms of item level fit, is better is likely due to the increase in the probabilities of answering the items correctly (i.e., proportion and counts are higher since it is a less strict criterion). In the case of items c and d for a perfect rating, as shown in the empirical plot (Fig 2 and 3), these two items revealed that the closer to the negative amount of theta (AIPWR), the more deviation between what is expected by the model and what is actually being estimated (i.e., confidence interval [in red lines] are far and above from the item characteristic curve [in blue]). Therefore, the observed probability of correctly answering the item at a low spectrum of AIPWR is higher than would be expected.”

**4. Your IRT parameter estimates have very large standard errors, in particular for the discrimination parameter in the 3-P model. Difficulties of estimating the discrimination parameter in 3-P models is a known problem, but I am still concerned. You may want to use a prior for the discrimination parameter in addition to a prior for the guessing parameter.**

**Answer:** We understand the concern regarding the SE for the 3-P model. To avoid an extra model (i.e., 3-P model with prior in guessing and discrimination), we added in the Results section the difficulties of estimating the discrimination exactly as you pointed out. Moreover, we gave extra reference about the parsimony rule involving estimating an extra-parameter.

Now, at page 15, it might be read as follows:

“It is important to note that the standard errors (SE) are larger than under IRT with three parameters, regardless of the adopted rating criterion as commonly described in the literature [40, 41].”

**5. I am also a bit worried about the magnitude for the guessing parameter for some items. For pure guessing, you would expect a guessing parameter around 1/12 = 0.08. Is the any theoretical that some items would have a lower asymptote of 0.3?**

**Answer:** The reviewer is correct about lower asymptote of 0.3. This is happening in the imperfect approach. We added an extra information about that point and we made a correction regarding our prior guessing for the imperfect approach, updating it to 3/12 due to the rating criteria where exact pitch responses and semitone deviations were considered correct (e.g.: aural stimulus: C; correct response: C, B or C#/Db). Based on that, we recalculated the prior guessing and the model’s parameters for imperfect rating. We thank you very much for the very attentive reading of our manuscript. Now, at line 207, it might be read as follows:

“Because the keyboard used to report the answers had 12 possibilities, the a priori value for the guessing parameter was 1/12 for the perfect rating; for imperfect rating criteria, it was 3/12 (see below the description for rating)”

**6. In choosing between latent class model, you argue that the improved fit of the models with more than 2 classes should be ignored, due to the complexity of these models. However, in comparisons between IRT and latent class models for the perfect approach, you suddenly argue that a difference in fit of the same magnitude is important and should be interpreted. You cannot have it both ways. If you compare e.g. a 3 class model with the IRT model, you get the following results:

AIC D AIC 3CL BIC D BIC 3CL SSABIC D SSABIC 3CL
Perfect 6988.887 6952.444 7082.150 7101.664 7018.640 7000.048

This comparison show no particular superiority of the IRT model in terms of fit. The same reasoning could be applied for a 4-CL or a 5-CL model. You may want to keep a 2-CL model in the comparisons for conceptual reasons, but you should include a latent class model with better fit (e.g. 3 classes or 4 classes). Based on the results I have seen, I would conclude that for the “perfect” approach, a latent class model with 3, 4, or 5 classes has equally good fit as an IRT model. For the “imperfect” approach, the IRT model seems clearly better.**

**Answer:** This was a crucial point. We revised all the Mplus output and we found some inconsistencies in our previous report for AICs and BICs. We are deeply sorry and grateful at that same time, as it made us revise all the numbers and, consequently, our results. Moreover, we added a new suggested modeling approach (hybrid solution). If the reviewer requires Mplus outputs, we would be happy to share with her/him. Now, at page 25 (line 406), our new results are describe as follows:

“Comparing the continuous, categorical, and hybrid solutions we concluded based on model fit information values that the continuous solution was the best solution for perfect approach, with lower BIC than the categorical and hybrid solutions. This indicates that the ability to recognize isolated pitches in different timbers and registers without reference is better modeled as a continuous ability when perfect rating approaches is considered in comparison with a categorical and hybrid model. Alternatively, the categorical solution demonstrated the best solution for the imperfect approach, with lower BIC than the continuous and hybrid solutions. This indicates that adopting flexibility in isolated pitch recognition without reference (with semitone deviations considered as correct) is better modeled as latent groups”

**7. You present the score distribution for one IRT model, presumably for the “perfect” approach (the text in lines 272-273 seems to have the numbering of figures wrong). The score distribution is clearly skewed. A large group of people seem to have the same level of ability to identify the pitch of a tone (i.e. no ability). This may pose a problem for standard IRT model estimation, since a normal distribution is assumed for the latent trait. For this reason, a better model for your data may be a latent mixture distribution model with 2 latent classes. Class one consist of persons who are not able to identify the pitch regardless of the stimulus. Class two consists of persons with at least some ability to identify pitch. Within class two responses might follow a 2-P IRT model. Such a model can be estimated in Mplus. It is fairly complex, but you may want to at least discuss it.**

**Answer:** We agree with the reviewer and we added the analysis of a hybrid model. The idea was really nice!

**8. With regards to the psychometric lingo, I would suggest that :
8a. “Psychometric” is better than “Psychometrical”
8b. “****Latent variable models” is better than “structural equation model”. SEM refers to a particular type of latent variable models, a type you do not use in your analysis.
8c. “Continuous” is better than “Dimensional”**

**Answer:** We accepted all the corrections and suggestions.

**Specific suggestions:
Line 24. I suggest “Through Latent Variable Models (LVM) we can evaluate consistency validity…”
Line 27. I suggest “… two LVM approaches: continuous latent variables (LV)…”
Line 37, I suggest “The phenomenon of absolute pitch (AP)…”
Line 65. It might be helpful to define relative pitch.
Line 115. Is there a reference regarding the use of a combination of AP and RP to identify pitches?
Line 159. I suggest “…continuous and categorical.”
Line 160. I suggest writing “The former approach, Item Response Theory (IRT), …”
Lines 166-170. I suggest writing: “a) An IRT model with two parameters for each stimuli:** **the discrimination parameter (also called parameter a), which describe the ability of this stimuli to distinguish between persons with low and high pitch identification ability and the item location parameter (also called parameter b), representing the level of pitch identification ability where you have 50% chance of correctly identifying the pitch of this stimulus.”
Lines 172+173. I suggest writing: “…** **is the probability of a person with very low pitch identification ability still correctly get a correct answer for a given stimulus.**

**Answer:** All the specific points were accepted and a new English-language editing service was contacted to double check spelling, coherence, and fluency.

**Reviewer #2: The ultimate aim of this research project is not explicitly stated, and it remains a little unclear. The authors have created a new test of assessing absolute pitch, and this was investigated using an item-response theory (IRT) approach and a latent class analysis (LCA) approach. These models were then compared to see which offered the best fit.
Although the foundation and rationale for the study seems reasonable, in that the authors wish to create a measure to determine whether individuals have the ability to identify absolute pitch, the applied methodologies are confusing and it should be better explained as to why they are appropriate.**

**Answer:** We thank the reviewer for the positive feedback and comments**.**

**• Latent Class Analysis categorises people into groups, under the assumption that the same thing is being measured for all people, by a standardised count, process, or measurement device/scale.
• IRT is used to determine whether a set of items are delivering a valid total score of an unobservable latent trait.
Thus it should be explained in more detail why the fit of these models should be compared as they look at different things. It should be emphasized that the categorisation of people relies on the measurement process being valid and stable, so in some sense LCA should not be considered until the measure has been validated.**

**Answer:** We improved information regarding CFA correcting for model fit and adding item fit indices as below required. For us, it was not clear what the reviewer#2 meant when s/he said about “**measurement process being valid and stable, so in some sense LCA should not be considered until the measure has been validated”.** Would it mean to conduct a CFA before LCA? CFA/IRT presumes homogeneity in parameter values across cases. LCA implies differences in parameter values, with each case participating in the different populations to a different degree. The hybrid modeling now added, CFA *with* LCA, accounts for heterogeneity by representing the data as a mixture of populations. Granting that LCA is primarily driven by differences in means rather than by differences in variances, we still would not trust parameter estimates (or fit assessment) from a CFA without LCA if we believed that a mixture model was best for the data. Here, we saw exactly that depending on the approach, the best way to model the data will change.

**For the IRT scale assessment approach, there are also many aspects that have been neglected. There is no indication of item fit. No investigation of response dependency. No assessment of reliability or targeting.**

**Answer:** This point was also raised by reviewer#1 and now, at page 9, it might be read as follows:

“To evaluate the model fit indices for IRT models, a Pearson chi-square test for categorical outcomes was used, with p-values higher than 0.05 indicators of a good fit. Item level fit was evaluated via Pearson’s X2 (S-X2) implemented in R package MIRT, as per Orlando and Thissen [34]”

**Was a single parameter model considered? - A single-parameter (Rasch) model would be appropriate** **for scale development and validation purposes, and for determining whether a total score from a set of items is a sufficient statistic to assess the level of a latent trait.**

**Answer:** We did not consider a single-parameter model leaving only 2 and 3 parameters. We did not find references discussing the superiority of 2/3 parameters modeling in comparison to single-parameter modeling for scale development and validation purposes. Based on the Deborah Bandalos 2018 book, on the chapter on Validity, it is stated that evidence based on internal consistency (i.e., commonly called as construct validity) are achieved by techniques involving latent variable approach not directly specifying the number of parameters to be estimated.

**Additionally, for the ‘imperfect approach’, it may be worth the authors considering a partial-credit model, where an exact pitch classification is awarded a score of 2, a semitone deviation is awarded 1, and all other pitches are scored 0.**

**Answer:** We understand the point raised by the reviewer and we understand the underlying idea of given this graded answer to the tasks. However, due to two main issues, we decide not to conduct it this way. Firstly, in the absolute pitch area, such scoring system (2, 1, 0) is not commonly adopted. Second, after the new added model (hybrid) plus other adds-on, we believe that the manuscript increased in its complexity. Adding a new modeling, which is only sensitive/meaningful for imperfect approach, will increase even more the complexity and length of our manuscript. This idea will be for sure further explored in future works.

**There are also some further issues within the manuscript that would need attention:
The model fit statistics are dubious, and there is no real interpretation of the fit statistics that are presented. Certainly a test with 1000 degrees of freedom will have no statistical power.**

**Answer:** We excluded the chi-square test for 1000 degrees of freedom and we improved the interpretability of the figure and new/old tables.

**In the manuscript, it is stated that items are centred around the 0 location, but there are no item locations reported below 0 – where are they centred?**

**Answer:** We are sorry for our imprecision. Now, at page 9, it might be read as follows:

“Under Maximum Likelihood estimator and using logit parameterization (theta), the constant 1.7 in the logit gives only an approximate closeness to the normal. The translation to IRT parameter values uses factor mean and factor variance to bring them to the N~(0,1) metric used in IRT”

**The pitch test is based across different musical instruments – have these instruments been calibrated? Has the pitch been externally verified in some way?**

**Answer:** The samples used were professionally recorded in a studio and are extremely accurate in terms of tuning, based on the A=440Hz standard. Since they are commercially used for a variety of soundtracks in cinema and television, their precision and quality is known among professional musicians. Nevertheless, the pitch of every sample used was checked before the battery test was assembled. The voice samples were recorded and edited by a professional studio technician. Two professional vocalists were assigned for the task. The pitches of the recorded voice samples were also checked (A=440Hz standard) and any deviation was corrected.

**Additionally, the manuscript is currently in need of a language edit and the Figures are incorrectly labelled.**

**Answer:** We agree with the reviewer that a language review in the whole manuscript was needed. We sent it to a new professional Editing-language editing service for a fully review. We deeply thank you for all the recommendations.

**6. PLOS authors have the option to publish the peer review history of their article (**[**what does this mean?**](https://journals.plos.org/plosone/s/editorial-and-peer-review-process#loc-peer-review-history)**). If published, this will include your full peer review and any attached files.


If you choose “no”, your identity will remain anonymous but your review may still be made public.


Do you want your identity to be public for this peer review? For information about this choice, including consent withdrawal, please see our** [**Privacy Policy**](https://www.plos.org/privacy-policy)**.**

Reviewer #1: Yes: Jakob Bue Bjorner

Reviewer #2: No


 **[NOTE: If reviewer comments were submitted as an attachment file, they will be attached to this email and accessible via the submission site. Please log into your account, locate the manuscript record, and check for the action link "View Attachments". If this link does not appear, there are no attachment files to be viewed.]


While revising your submission, please upload your figure files to the Preflight Analysis and Conversion Engine (PACE) digital diagnostic tool,** [**https://pacev2.apexcovantage.com/**](https://pacev2.apexcovantage.com/)**. PACE helps ensure that figures meet PLOS requirements. To use PACE, you must first register as a user. Registration is free. Then, login and navigate to the UPLOAD tab, where you will find detailed instructions on how to use the tool. If you encounter any issues or have any questions when using PACE, please email us at** [**figures@plos.org**](mailto:figures@plos.org)**. Please note that Supporting Information files do not need this step.**
