# Supplementary material for: A new approach to measuring absolute pitch on a psychometric theory of isolated pitch perception: Is it disentangling specific groups or capturing a continuous ability?
Source: PLoS One. 2021 Feb 22;16(2):e0247473. doi: 10.1371/journal.pone.0247473 (PMC7899369; doi:10.1371/journal.pone.0247473)
Supplement: Attachment — Submitted filename: Response_to_Reviewers.docx [file pone.0247473.s004.docx]

PONE-D-20-04410R1
A new Approach to Measuring Absolute Pitch on a Psychometric Theory of Isolated Pitch Perception: Is it Disentangling two Groups or Capturing a Continuous Ability?
PLOS ONE

Dear Dr. Germano,

Thank you for submitting your manuscript to PLOS ONE. After careful consideration, we feel that it has merit but does not fully meet PLOS ONE’s publication criteria as it currently stands. Therefore, we invite you to submit a revised version of the manuscript that addresses the points raised during the review process.

Thank you for your careful revision. I agree with the reviwers that the manuscript has improved. The comments from the reviewers on the revised version illustretes that more work is needed. I agree with comment that the manuscript is currently too long and too difficult to read. I am also concerned about the results you obtain from the 3PL model. The very large standard errors, and the large guessing parameters makes me worry that these results cannot be trusted. One way to make the manuscript better would be to put less emphasis on these results.

Please carefully consider all the points raised in the attached reviews and submit your revised manuscript by Oct 23 2020 11:59PM. If you will need more time than this to complete your revisions, please reply to this message or contact the journal office at plosone@plos.org. When you're ready to submit your revision, log on to https://www.editorialmanager.com/pone/ and select the 'Submissions Needing Revision' folder to locate your manuscript file.

Please include the following items when submitting your revised manuscript:

- A rebuttal letter that responds to each point raised by the academic editor and reviewer(s). You should upload this letter as a separate file labeled 'Response to Reviewers'.
- A marked-up copy of your manuscript that highlights changes made to the original version. You should upload this as a separate file labeled 'Revised Manuscript with Track Changes'.
- An unmarked version of your revised paper without tracked changes. You should upload this as a separate file labeled 'Manuscript'.

If you would like to make changes to your financial disclosure, please include your updated statement in your cover letter. Guidelines for resubmitting your figure files are available below the reviewer comments at the end of this letter.

If applicable, we recommend that you deposit your laboratory protocols in protocols.io to enhance the reproducibility of your results. Protocols.io assigns your protocol its own identifier (DOI) so that it can be cited independently in the future. For instructions see: <http://journals.plos.org/plosone/s/submission-guidelines#loc-laboratory-protocols>

We look forward to receiving your revised manuscript.

Kind regards,

Karl Bang Christensen, Ph.D.
Academic Editor
PLOS ONE

[Note: HTML markup is below. Please do not edit.]

Reviewers' comments:

Reviewer's Responses to Questions

**Comments to the Author**

1. If the authors have adequately addressed your comments raised in a previous round of review and you feel that this manuscript is now acceptable for publication, you may indicate that here to bypass the “Comments to the Author” section, enter your conflict of interest statement in the “Confidential to Editor” section, and submit your "Accept" recommendation.

Reviewer #1: (No Response)

Reviewer #2: All comments have been addressed

2. Is the manuscript technically sound, and do the data support the conclusions?

The manuscript must describe a technically sound piece of scientific research with data that supports the conclusions. Experiments must have been conducted rigorously, with appropriate controls, replication, and sample sizes. The conclusions must be drawn appropriately based on the data presented.

Reviewer #1: No

Reviewer #2: Yes

3. Has the statistical analysis been performed appropriately and rigorously?

Reviewer #1: Yes

Reviewer #2: I Don't Know

4. Have the authors made all data underlying the findings in their manuscript fully available?

The [PLOS Data policy](http://www.plosone.org/static/policies.action#sharing) requires authors to make all data underlying the findings described in their manuscript fully available without restriction, with rare exception (please refer to the Data Availability Statement in the manuscript PDF file). The data should be provided as part of the manuscript or its supporting information, or deposited to a public repository. For example, in addition to summary statistics, the data points behind means, medians and variance measures should be available. If there are restrictions on publicly sharing data—e.g. participant privacy or use of data from a third party—those must be specified.

Reviewer #1: No

Reviewer #2: Yes

5. Is the manuscript presented in an intelligible fashion and written in standard English?

PLOS ONE does not copyedit accepted manuscripts, so the language in submitted articles must be clear, correct, and unambiguous. Any typographical or grammatical errors should be corrected at revision, so please note any specific errors here.

Reviewer #1: No

Reviewer #2: Yes

**Answer: The first and second manuscript was revised by a professional English-language editing service. The new version of the manuscript was also fully revised by another professional English-language editing service.**

6. Review Comments to the Author

Please use the space provided to explain your answers to the questions above. You may also include additional comments for the author, including concerns about dual publication, research ethics, or publication ethics. (Please upload your review as an attachment if it exceeds 20,000 characters)

Reviewer #1: Thanks for the responses to my previous comments. I find that your revisions have clarified the data analyses, but there is still some way to go.
You have two scoring options for assessment of isolated pitch perception: a. Perfect: only the correct note is classified as a correct response, b. Imperfect: including the half-note below and above. You compare 1. IRT analyses using either a 2-PL or a 3-PL model, 2. Latent class analyses, 3. Mixture model analyses. You conclude that perfect scoring is best fitted using an IRT model, while imperfect scoring is best fitted with a two-class latent class model.

1. I understand that your choice of model is guided by the global indices such as BIC and AIC. However, from a conceptual point of view, this conclusion does not make sense to me. If you conclude that isolated pitch perception with perfect scoring represent a continuous latent trait, how could the use of a less stringent scoring criterion suddenly change this ability to two latent classes? It seems to me that this interpretation of results is theoretically incoherent. I think you need to discuss this and make an overall interpretation whether isolated pitch perception is best considered a continuous trait or two latent classes.

**Response: Thank you for your observation. AP literature shows different methods to measure AP ability, which led to the two rating criteria (perfect and imperfect approaches) adopted in our test. To clarify, we conducted a brief review of the two methods (see table below), showing that both methods are largely used universally. Our aim was not to directly compare these two approaches. Rather, we aimed to evaluate how they might influence the decision of the best model for isolated pitch recognition tasks (line 111), once they are both used arbitrarily in the literature. One of our hypotheses was that different measurement methods lead to different results that reflect different theoretical perspectives. This is a methodological issue that is not adequately addressed by AP literature. Our results corroborate this hypothesis, as the two rating approaches showed distinct underlying models (a continuous trait for the perfect approach and two latent classes for the imperfect approach). Also, our aim was to contribute to a better theoretical understanding of AP ability, showing that different rating criteria greatly influences test results and the underlying model. We added the imperfect result in the abstract to highlight this duality regarding AP measurement (line 37). In the conclusion, we added our contribution to the AP literature (line 511). In the discussion, we added an observation regarding the change from a continuous model to a two latent classes model resulting from the adoption of a less stringent scoring criterion, as mentioned (line 466).**

2. Related to the discussion above, it seem to me that the latent class solution for imperfect scoring has some unfortunate interpretation. Even the best group has only a 67% chance of getting item i right ad 72% change of getting item j right within +/- a half tone. This does not concur with the common understanding of absolute pitch. Maybe the absolute pitch group is only a subgroup within the current best latent class. In that case, you may need more than two latent classes. Please discuss whether your current model is theoretically plausible.

**Response:**

**We thank the reviewer for raising this issue.**

**AP possessors do not have exceptional pitch acuity. Absolute pitch is neither ‘absolute’ nor ‘perfect’ in the ordinary uses of those words (Levitin e Rogers, 2005). AP literature shows that it is quite common for AP possessors to make semitone errors in pitch judgments (Rogers and Levitin, 2005; Miyazaki, 1988; Baggaley, 1974; Brady, 1970). This results in semitone errors being considered as a partial or totally correct answer in some tests (Baharloo et al., 1998; Athos et al., 2007; Schulze et al., 2009). In our study, under the imperfect approach, the better group had a 67% chance of getting item *i* right and a 72% chance of getting item *j* right. These results are consistent with previous research using the imperfect approach, considering semitone errors as ½ correct (73% correct), not allowing for semitone errors and 80% correct allowing for semitone errors, [Li (2020]).**

**Therefore, contrastingly to what Reviewer#1 mentioned, and as described by Levitin and Rogers (2005), page 28,** “... ‘absolute’ refers to judgments established independently, rather than by comparison. The terms ‘absolute’ and ‘perfect’ both imply in the lay mind a level of precision not typically present in AP possessors, who frequently make octave errors (confusing tones that are half or double the frequency), and semitone errors (confusing tones that are 6% apart) [Levitin, D.J. (1999) Absolute pitch: Self-reference and human memory. International Journal of Computing Anticipatory Systems 4, 255–266–Miyazaki, K. (1988) Musical pitch identification by absolute pitch possessors. Percept. Psychophys. 44, 501–512]. Like most human traits, AP is not an all-or-none ability, but rather, exists along a continuum [Levitin, D.J. (2004) L’oreille absolue. L’Annee´ Psychologique 104, 103–120; Levitin, D.J. (1999) Absolute pitch: Self-reference and human memory. International Journal of Computing Anticipatory Systems 4, 255–266; Deutsch, D. (2002) The puzzle of absolute pitch. Curr. Dir. Psychol. Sci. 11, 200–204; Vitouch, O. (2003) Absolutist models of absolute pitch are absolutely misleading. Music Perception 21, 111–117]. Self-identified AP possessors score well above chance (which would be 1 out of 12, or 8.3%) on AP tests, typically scoring between 50 and 100% correct [Miyazaki, K. (1988) Musical pitch identification by absolute pitch possessors. Percept. Psychophys. 44, 501–512], and even musicians not claiming AP score up to 40% [Lockhead, G.R. and Byrd, R. (1981) Practically perfect pitch. J. Acoust. Soc. Am. 70, 387–389]. Still, even those who score better than 90% show similar discrimination thresholds to, and are typically no better than, other musicians at noticing when one tone is out of tune with respect to another [Burns, E.M. and Campbell, S.L. (1994) Frequency and frequency-ratio resolution by possessors of absolute and relative pitch: Examples of categorical perception? J. Acoust. Soc. Am. 96, 2704–2719; Levitin, D.J. (1999) Absolute pitch: Self-reference and human memory. International Journal of Computing Anticipatory Systems 4, 255–266]. Clearly, there is nothing ‘perfect’ about AP; rather AP is the ability to place or produce tones within nominal categories.

**This discussion was included in line 96. We also conducted a three latent class analysis, but the two classes solution was best, as presented in line 379. If there were volunteers who were near infallible in isolated pitch recognition tasks in our experiment, they could not be identified as a separate group among the participants. This discussion was added in line 466.**

3. For perfect scoring, you find that an IRT model represent the best model for the data. However, for perfect scoring, evaluation of item fit finds significant misfit for item c and item d. This suggests problems for these two items as indicators of the latent trait. This is not discussed, but should be. This plots and fit tests do not suggest that a 3-PL model provides a better fit to the data than a 2-PL model.

**Response: We thank the Reviewer for this suggestion. We agree with the Reviewer, especially considering the parsimony principle. We modified our text, accordingly stating that the 2-PL is the best solution for both ratings under the IRT approach.**

4. I continue having concerns about your 3-PL model. The discrimination parameter is very high for item b and so it the guessing parameter. Also, the standard errors of the discrimination parameter are high. The BIC suggest that the 2-PL model might be the best. You should conclude which model you regard as the final model.

**Response: Thank you for your considerations. The 2-PL model was concluded as the best model. The text modification was made in line 379-384 and in line 461.**

5. You aim to develop a test for isolated pitch recognition. I assume that part of this development is to decide whether your test is best scored using the perfect or the imperfect approach. I think you should provide your recommendation and the reasoning behind it.

**Response: As previously discussed, our aim was not to compare these two score approaches (because the models are not nested). We sought instead to evaluate how they might influence the decision of the best model for isolated pitch recognition tasks (line 112). As score approaches reflect different theoretical perspectives, we believe our results contribute to a better theoretical understanding of AP ability, showing that different rating criteria greatly influences test results and the nature of the underlying latent variable. Also, we cannot formally compare imperfect and perfect approaches in regarding superiority because they are not nested models (line 453).**

**The test comprised auditory stimuli and was collectively applied. Volunteers were given a keyboard drawn on paper on which they marked the answer that they thought was correct. This procedure allowed us to standardize the evaluation procedure. The correction was conducted in two ways (perfect and imperfect approaches), according to the literature.**

**Given below are examples of studies that use these two approaches:**

| **Paper** | **Authors** | **Periodic** | **Number of citations** | **Perfect/Imperfect** |
| --- | --- | --- | --- | --- |
| Auditory Stroop and Absolute Pitch: An fMRI Study | Katrin Schulze, Karsten Mueller, and Stefan Koelsch | Human Brain Mapping  2013 | 42 | *Imperfect* (answers within one semitone  were regarded as a correct answer) |
| Intracortical Myelination in Musicians  With Absolute Pitch: Quantitative  Morphometry Using 7-T MRI | Seung-Goo Kim and Thomas R. Kn€osche | Human Brain Mapping  2016 | 22 | *Imperfect* (An error with a semitone was considered as a correct response) |
| Increased Volume and Function of Right Auditory Cortex as a Marker for Absolute Pitch | Martina Wengenroth, Maria Blatow, Armin Heinecke, Julia Reinhardt,  Christoph Stippich, Elke Hofmann and Peter Schneider | Cerebral Cortex  2014 | 80 | *Imperfect* (for semitone errors 0.5 point was  Accredited) |
| Gray and  White Matter  Anatomy of Absolute  Pitch Possessors | Anders Dohn, Eduardo A. Garza-Villarrea, M. Mallar Chakravarty,  Mads Hansen, Jason P. Lerch, and Peter Vuust | Cerebral Cortex  2015 | 49 | *Imperfect* (Participants were given  ¾ point for each error of a semitone) |
| Perceiving pitch absolutely: Comparing absolute and relative pitch possessors in a pitch memory task | Katrin Schulze, Nadine Gaab and Gottfried Schlaug | BMC Neuroscience  2009 | 102 | *Imperfect* (answers within one semitone of the sented pitch as a correct answer) |
| The Neurocognitive Components of Pitch Processing: Insights from Absolute Pitch | Sarah J. Wilson, Dean Lusher, Catherine Y. Wan,  Paul Dudgeon and David C. Reutens | Cerebral Cortex  2009 | 101 | *Perfect* (semitone  errors were coded as incorrect for all participants) |
| Absolute Pitch and the P300 Component of the Event-Related Potential: An Exploration of  Variables That May Account for Individual Differences | Laura Renninger, Roni Granot, Emanuel Donchin | Music Perception  2003 | 34 | *Imperfect* (credit was given to subject was came within 1 semitone of the correct pitch) |
| Absolute Pitch—Functional Evidence of Speech-Relevant Auditory Acuity | Mathias S. Oechslin, Martin Meyer and Lutz Jäncke | Cerebral Cortex  2010 | 125 | *Perfect* (the semitone errors were taken as incorrect to increase the  discriminatory power) |
| Absolute Pitch and Planum Temporale | Julian Paul Keenan, Ven Thangaraj, Andrea Halpern, Gottfried Schlaug | NeuroImage  2001 | 354 | *Imperfect* (we regarded a response within 1/2 tone  difference of the presented tone as a correct response) |
| Absolute Pitch: An Approach for Identification of Genetic and  Nongenetic Components | Siamak Baharloo, Paul A. Johnston, Susan K. Service, Jane Gitschier,  and Nelson B. Freimer | Am. J. Hum. Genet.  1998 | 350 | *Imperfect* (we decided  to score a full point for semitone errors made by individuals >45 years of age) |
| A Distribution of Absolute Pitch Ability as Revealed  by Computerized Testing | Patrick Bermudez And Robert J. Zatorre | Music Perception  2009 | 71 | *Perfect* (In the percent correct score, only exactly correct responses are counted (0 semitone  deviation) |
| Absolute pitch is associated with a large auditory digit span: A clue to its genesis (L) | Diana Deutsch and Kevin Dooley | J. Acoust. Soc. Am.  2013 | 31 | *Perfect* (not allowing for semitone errors). |
| Absolute pitch among American and Chinese conservatory  students: Prevalence differences, and evidence  for a speech-related critical period | Diana Deutsch, Trevor Henthorn, Elizabeth Marvin, HongShuai Xi | J. Acoust. Soc. Am.  2006 | 317 | *Perfect* and *Imperfect* (no semitone errors allowed and semitone errors allowed). |
| Dichotomy and perceptual distortions in absolute  pitch ability | Alexandra Athos, Barbara Levinson, Amy Kistler, Jason Zemansky, Alan Bostrom, Nelson Freimer,  and Jane Gitschier | PNAS  2007 | 130 | *Imperfect* (partial  [3/4 point] credit for an answer deviating by one semitone |
| Effects of Musical Training and Absolute Pitch on a Pitch Memory Task: an Event-related  Potential Study | Edwin C Hantz, Kelley G. Kreilick, Amy L. Braveman, Kenneth P. Swartz | Psychomusicology  1995 | 18 | *Perfect* (no points were given for any  other answers; that is, no points were awarded for near hits half-step errors). |
| The effects of timbre on  absolute pitch judgment | Xiaonuo Li | Psychology of Music  2020 | 0 (new research) | *Perfect* and *Imperfect* (no semitone errors allowed and semitone errors allowed). |
| Absolute Pitch as an Inability: Identification of musical interval in a tonal context | Ken’ichi Miyazaki | Musical Perception  1993 | 101 | *Imperfect* (semitone errors are counted as correct) |
| Perfect Pitch | Joseph Profita and T. George Bidder | American Journal of Medical Genetics  1988 | 239 | *Perfect* (Subjects were defined as having perfect pitch if they were able to identify 90% or  more of the total number of tones). |
| Absolute Pitch and the P300 Component of the Event-Related Potential: An Explanatory of Variables that may Account for Individuals Differences | Laura Bischoff Renninger, Roni Granot, Emanuel Donchin | Music Perception  2003 | 34 | *Imperfect* (credit was given to subject who came within 1 semitone of the correct pitch) |
| Absolute Pitch: Effects of Timbre on Note-Naming Ability | Patrícia Vanzella, E. Glenn Schellenberg | PLoS ONE  2010 | 50 | *Imperfect* (semitones errors were considered as correct) |
| Effects of musical training and absolute pitch ability  on event-related activity in response to sine tones | John W. Wayman, Robert D. Frisina and Joseph P. Walton | Acoustical Society of America  1992 | 78 | *Imperfect* (half-step errors were given half credit) |
| How Stable is Pitch Labeling Accuracy in Absolute Pitch Possessors? | Wilfried Gruhn, Reet Ristmägi, Peter Schneider, Arun D'souza, Kristi Kiilu | Empirical Musicological Review  2018 | 1 | *Imperfect* (For semitone errors 0.5 point was accredited) |
| Absolute pitch memory: Its prevalence among musicians  and dependence on the testing context | Yetta Kwailing Wong & Alan C.N. Wong | Psychon Bull Rev  2013 | 6 | *Perfect* (discriminating  between neighboring semitones [e.g., treating a “C” as a “C#”] are regarded as errors)  (Takeuchi & Hulse, 1993;  Zatorre, 2003). |
| Multiple coding strategies in the retention of musical tones by possessors of absolute pitch | Robert J. Zatorre, Christine Beckett | Memory & Cognition  1989 | 113 | *Perfect* (0% correct by semitone transposition) |

6. The paper is a long and complex read because some many combinations of options are examined. You would increase readability by focusing the main paper on what you consider the best solution and present other options as supplemental analyses. For example, you could focus on perfect scoring, use the 2-PL model as your IRT model to be compared with a latent class and a mixture analysis. Other options could be alluded to briefly and results for these other models could be presented in a web appendix. I think such an approach would make your paper much more readable.

**Response: Thank you for your comment. We agree that this paper is a long and complex read. Nevertheless, all these analyses are of paramount importance to researchers in the AP area. We believe that the audience and the impact of this paper will be expanded if these two scoring approaches commonly cited and used in AP literature are included in the main text. Please see the table above for your reference where we mapped the manuscripts, number of citations, the adopted approaches, and other bibliometric features. Applying psychometrics to AP may guide future studies, since our work provides different analytical perspectives for a set of stimuli commonly used to track AP. That is, if our paper addresses these two different approaches (imperfect versus perfect), it will be increasingly cited in the future due to the novel AP research. As mentioned above, the two score approaches reflect different theoretical perspectives which are commonly adopted. We believe that this full comparative and statistical discussion will contribute to a better theoretical understanding of AP ability, showing that different rating criteria greatly influences test results and the underlying model.**

7. You write that the differences in item difficult and discrimination poses difficulties for a simple sum score approach. However, items may be summed without problems even if they have widely different item difficulty. For example, in the Rasch model (where all items have the same discrimination, but may differ in item difficulty) the sum score is a sufficient statistics for the latent trait. So the real issue is whether the items very so much in item difficulty that a simple sum is inappropriate. For the 2-PL model and perfect scoring, I do not think this is the case. Item discrimination varies between 1.2 and 1.9, not a dramatic variation. It is possible that a Rasch (i.e. 1-PL model) may fit these items. Please revise this discussion.

**Response: We agree with the Reviewer. The process of parceling (summing or averaging items) has shown its robustness based on the law of large numbers and aggregation principles (see Little et al., 2002). The sentence has been removed from the text.**

**Little, T. D., Cunningham, W. A., Shahar, G., & Widaman, K. F. (2002). To parcel or not to parcel: Exploring the question, weighing the merits. Structural equation modeling, 9(2), 151-173.**

8. While the analyses seems to be well done, the interpretation and discussion of results could use input from English language researchers with psychometric / IRT expertise. Some description of the models is not well structured (e.g. the discussion of IRT models on lines 205-219). Also, while most of the paper is well written, some parts of the psychometric discussion still deviates for the normal language of the field, e.g. in the abstract [my suggestions in square brackets]:

“We decided to adopt a psychometric perspective, approaching AP as a latent trait. Via Latent Variable Model (LVM) we can provide [evaluate] consistency and validity for a measurement [measure] to test for AP ability. A total of 783 undergraduate music students took part in the test. The battery test [test battery] consisted of 10 isolated pitches.”

**Response: Thank you for your suggestions. We changed the words in the abstract and in the lines indicated.**

Reviewer #2: I would like to thank the authors for responding to the reviewers’ requests and making appropriate amendments to their paper. They have clearly invested time and effort into this, and I believe that the manuscript is now presented better and is much clearer in terms of the purpose of the paper and the process that has been carried out.

There are a few additional amendment suggestions that I have, and these are provided below:

I would suggest that the title is amended to state ‘specific groups’ or ‘ability groups’ rather than ‘two groups’. As you are using LCA, it is not known a priori how many latent classes will be identified.

**Response: Thank you for the suggestion. We changed “two groups” to “specific groups”.**

The Intro reads well, provides good background and makes sense. However, this is an exploratory study, to see how the items in the AP test work among the group tested. Do the items work together to form a measure of an underlying latent continuum (IRT)? Or do they work better as a set of indicator items that can classify people into groups (LCA)? I would suggest that this may be clarified for readers if the authors were to provide a statement in both the abstract and the introduction to state that this is an exploratory data modelling study, to determine which type of model best fits the data for the tested sample.

**Response: Thank you for your comments. AP literature indicates different approaches to rating the stimuli of isolated pitch perception, which led to the two rating criteria (the perfect and imperfect approaches) adopted in our test. Our aim was not to compare these two approaches, but to evaluate how they might influence the decision of the best model for isolated pitch recognition tasks within each approach (line 112). One of our hypotheses was that different approaches lead to different results. It is important to note that both approaches are used in AP research and, to our knowledge, this psychometric issue was not adequately addressed by AP literature. Our results corroborate this hypothesis, as the two rating approaches showed the distinct nature of latent variables (a continuous trait for the perfect approach and two latent classes for the imperfect approach). Also, our aim was to contribute to a better theoretical understanding of AP ability, showing that different rating criteria greatly influence test results and how the latent variable might be measured. We agree that this issue needed further clarification in the manuscript. Therefore, we included more information in lines 96, 466 and 509.**

For the IRT analysis, there is still no test of local dependency among the items. This is perhaps unnecessary for the purpose of the current study, but perhaps it could be identified as a potential limitation, or the authors could suggest that it could be assessed in future work if a latent trait IRT approach is pursued further.

**Response: We agree with the Reviewer. We added the following: “Future studies may investigate more detailed elements of psychometrics as local dependency for each of the models (IRT and LCA), invariance testing per sex, time of studies, and played instruments” (lines 496 – 498)**

The authors state ‘Under Maximum Likelihood estimator and using logit parameterization (theta), the constant 1.7 in the logit gives only an approximate closeness to the normal. The translation to IRT parameter values uses factor mean and factor variance to bring them to the N~(0,1) metric used in IRT.’ To clarify this for the reader, I would suggest that the authors might also add a sentence to state that this means that the IRT analysis is centred on the person sample being at 0 logits, and that the item difficulty parameters are provided relative to this.

**Response: We thank the Reviewer for this comment. It now reads in line 216 as: “The factor is assumed to be normally distributed being the mean fixed at zero and factor variance at 1. That is, the IRT analysis is centered on the person sample being at 0 logits, and that the item difficulty parameters are provided relative to this”**

A few additional very minor corrections are as follows:

Line 189 states that MPlus is used. R also needs to be added here.

**Response: Thank you for the observation. The R program was included.**

Line 287. I believe this should say difficulty rather than discrimination.

**Response: Thank you. The word has been changed.**

Line 409. Should be timbres rather than timbers.

**Response: You are correct. The revision has been made.**

A list of abbreviations would also be useful, so that the reader can refer back to them without scrolling through all of the manuscript to find the relevant abbreviation.

**Response: Thank you for your suggestion. The list of abbreviations was included as Supporting Information.**

7. PLOS authors have the option to publish the peer review history of their article ([what does this mean?](https://journals.plos.org/plosone/s/editorial-and-peer-review-process#loc-peer-review-history)). If published, this will include your full peer review and any attached files.


If you choose “no”, your identity will remain anonymous but your review may still be made public.


**Do you want your identity to be public for this peer review?** For information about this choice, including consent withdrawal, please see our [Privacy Policy](https://www.plos.org/privacy-policy).

Reviewer #1: **Yes:** Jakob Bue Bjorner

Reviewer #2: No

[NOTE: If reviewer comments were submitted as an attachment file, they will be attached to this email and accessible via the submission site. Please log into your account, locate the manuscript record, and check for the action link "View Attachments". If this link does not appear, there are no attachment files.

While revising your submission, please upload your figure files to the Preflight Analysis and Conversion Engine (PACE) digital diagnostic tool, <https://pacev2.apexcovantage.com/>. PACE helps ensure that figures meet PLOS requirements. To use PACE, you must first register as a user. Registration is free. Then, login and navigate to the UPLOAD tab, where you will find detailed instructions on how to use the tool. If you encounter any issues or have any questions when using PACE, please email PLOS at [figures@plos.org](mailto:figures@plos.org). Please note that Supporting Information files do not need this step.
